# 119 Single Center Central Line Associated Bloodstream Infection with Implementation of Central Venous Catheter Policy

**DOI:** 10.1093/jbcr/irae036.118

**Published:** 2024-04-17

**Authors:** Scott Olehnik, Kaitlyn M Libraro, Sylvia H Dao, Abraham Houng

**Affiliations:** New York Presbyterian Weill Cornell Medical Center, New York, New York; New York - Presbyterian / Weill Cornell, new York, NY; NY Presbyterian/ Weill Cornell Medical College, New York, NY; New York Presbyterian Weill Cornell Medical Center, New York, New York; New York - Presbyterian / Weill Cornell, new York, NY; NY Presbyterian/ Weill Cornell Medical College, New York, NY; New York Presbyterian Weill Cornell Medical Center, New York, New York; New York - Presbyterian / Weill Cornell, new York, NY; NY Presbyterian/ Weill Cornell Medical College, New York, NY; New York Presbyterian Weill Cornell Medical Center, New York, New York; New York - Presbyterian / Weill Cornell, new York, NY; NY Presbyterian/ Weill Cornell Medical College, New York, NY

## Abstract

**Introduction:**

Central venous line (CVL) associated bloodstream infection (CLABSI) occurs at a much higher rate in burn patients. The incidence of CLABSI in burns has been reported to be two to three times that of other critical care patients. Many burn centers have their own central line policy; however, the policies are not consistent across different burn centers, and no guidelines have been published. In this study, we analyzed our institution’s CLABSI rate retrospectively after implementing a strict CVL policy.

**Methods:**

Retrospective chart review was performed on a single institution burn center in the calendar year of 2022 and 2017-2018. The time period was chosen to account for any discrepancies due to the COVID-19 pandemic. Pediatric patients were excluded. Following data were collected: age, burn size, mechanism, length of hospital stays, CVL days, blood stream infection, and organism. CLABSI bundle information was also collected: optimal site, aseptic maintenance, timely removal, and education. This data was compared with pre-implementation data in 2017-2018. This was a quality improvement project.

**Results:**

There were 420 patients in the study period in 2022. There were 56 patients with CVL for a total of 441 line-days. Of all the patients with CVL, there was only one CLABSI, with the incident of CLABSI being 2.27 per 1000 line days in 2022. The CLABSI standard infection ratio (SIR) was calculated to be 0.08 (SD 0.11). The decrease in CLABSI was statistically significant when compared with pre-intervention SIR of 0.77 in 2017-2018 (SD 0.169), p=0.02. The absolute incident of CLABSI went from 5 to 1, p=0.01.

**Conclusions:**

This institution’s CVL policy consists of quantifying the line into burn CVL or non-burn CVL. Burn CVL is any line within 5cm of a burn wound. Burn CVL is changed every 3 days, and non-burn CVL is changed every 7 days. The policy allows for one over-wire CVL exchange. A new site is needed after the over-wire exchange. From the CLABSI data, this policy decreased the CLABSI SIR and absolute CLASI incidents. A multi-center implementation of the policy is needed to provide a more robust data and help combat CLABSI in the burn unit.

**Applicability of Research to Practice:**

Directly applicable.

